# *Candida auris*: An emerging pathogen “incognito”?

**DOI:** 10.1371/journal.ppat.1007638

**Published:** 2019-04-08

**Authors:** Jeniel E. Nett

**Affiliations:** Departments of Medicine and Medical Microbiology & Immunology, University of Wisconsin, Madison, Wisconsin, United States of America; Geisel School of Medicine at Dartmouth, UNITED STATES

## What is unique about *Candida auris*?

The emerging fungal pathogen *Candida auris* is causing outbreaks of invasive disease in healthcare facilities around the world [1–4]. Isolates often exhibit resistance to multiple drug classes, and invasive disease carries an astonishingly high mortality rate, approaching 60% [5]. The initial description of *C*. *auris* arose following the isolation of a novel species from the ear canal of a patient in a Japanese hospital [2]. Since this report in 2009, cases have subsequently appeared in hospitals in South Korea, India, the Middle East, South Africa, and South America [6–10]. More recent reports reveal the spread of *C*. *auris* to North American and European healthcare facilities [3, 11]. Genomic analysis shows that the circulating strains cluster into distinct clades, which appear to have emerged independently [5]. Retrospective analyses of *Candida* isolates collected from international sites in the decades leading to 2009 uncovered only rare cases of *C*. *auris*, confirming the recent emergence of this species [5, 10].

*C*. *auris* is the first fungal pathogen categorized as a public health threat due to its ability to readily colonize skin, spread rapidly among patients, and cause severe disease [3–5, 12]. The efficient person-to-person transmission observed for *C*. *auris* is striking, because candidiasis caused by other species typically arises from the patient’s own microbiome, often from the gastrointestinal tract. However, unlike other *Candida* spp., *C*. *auris* does not appear to efficiently colonize the gastrointestinal tract, presumably due to its poor growth under anaerobic conditions [13]. An additional factor contributing to the spread of *C*. *auris* is the propensity of the species to persist on the surfaces of hospital rooms and on medical devices [14, 15]. With these barriers to nosocomial control, *C*. *auris* continues to spread. In an area where it first emerged, *C*. *auris* now accounts for nearly 20% of *Candida* bloodstream isolates, surpassing that of *C*. *albicans*, which is typically the most common species [16].

## Who develops *C*. *auris* infection?

*C*. *auris* can cause invasive disease involving a variety of clinical niches [3, 5]. The most frequently reported site is the bloodstream, with isolation from the urinary or respiratory tract close behind [5]. Despite the high frequency of most *Candida* species to cause oral or esophageal disease, *C*. *auris* is not frequently reported at these sites. A study by Pathirana and colleagues shed light on one reason for this apparent void. They examined activity of a salivary cationic peptide, histatin 5, against *C*. *auris* and found the vast majority of isolates to be highly sensitive, particularly those exhibiting antifungal resistance [17]. Another intriguing observation is that a common risk factor for invasive candidiasis, neutropenia, has not been reported for *C*. *auris* infection, because this is a common risk for other *Candida* spp. [5, 6]. Invasive disease in the absence of neutropenia suggests that the host neutrophil response may not be adequate for control of *C*. *auris*.

Pursuits to uncover the traits of patients with invasive *C*. *auris* disease have revealed a common theme. These patients have often undergone multiple medical interventions, including surgical procedures, mechanical ventilation, vascular catheterization, and gastrostomy tube placement [5, 18]. The widespread use of artificial devices evokes the question of surface-associated biofilms. Although isolates of *C*. *auris* can form biofilms in vitro, they appear to grow to a biomass less than that of *C*. *albicans* [19, 20]. However, similar to biofilms formed by other *Candida* spp., *C*. *auris* biofilms exhibit drug resistance beyond that observed during planktonic growth [19]. How biofilm formation by *C*. *auris* may influence immunity is unknown. However, biofilms formed by other *Candida* species resist phagocytic killing [21–24].

## How do animal models mimic *C*. *auris* infection in patients?

Several groups have explored the pathogenicity of *C*. *auris*, employing a variety of model systems [11, 19, 25–28]. Initial reports utilized an invertebrate moth larvae (*Galleria mellonella*) model of invasive candidiasis [11, 19]. These studies examined *C*. *auris* strains collected in the United Kingdom and found them to be considerably more virulent than other nonfilamentous *Candida* isolates. A subset of *C*. *auris* exhibited pathogenicity similar to even the most virulent species, *C*. *albicans* and *Candida tropicalis* [11]. Of note, the more virulent *C*. *auris* isolates grew in nonaggregative forms, in contrast to the less pathogenic strains that formed large aggregates. As these nonaggregative forms also tended toward more robust biofilm formation, it is intriguing to speculate clinical relevance for this phenotype [19].

Investigation of *C*. *auris* in murine models of invasive candidiasis have included animals with both compromised and intact immunity [25–27]. Using BALB/c mice with cyclophosamide-induced neutropenia, Ben-Ami and colleagues examined the virulence of a *C*. *auris* strain collected in Israel [25]. This strain exhibited virulence beyond that of the closely related *C*. *haemulonii*, which was nonvirulent. However, *C*. *auris* was significantly less virulent than *C*. *albicans*, in which animals succumbed more rapidly (1 versus 4 days), and 10-fold higher fungal burdens were noted in the kidney [25]. Examination of the kidneys of mice that had succumbed to *C*. *auris* infection revealed the formation of aggregates, which the authors propose may serve as a mode of immune evasion [25]. If and how these renal aggregates relate to the aggregative forms found in vitro remains unclear [11, 19]. In contrast, a second investigation examining 2 *C*. *auris* isolates from India in an immunocompetent IRC murine model of disseminated candidiasis revealed survival similar to *C*. *albicans* [27]. In a third investigation using BALB/c mice, Wang and colleagues compared the virulence of an isolate of *C*. *auris* from China to *C*. *albicans* and found *C*. *albicans* to be more virulent [26]. In this invasive candidiasis model, mice infected with *C*. *albicans* died by day 6 whereas the *C*. *auris*-infected animals survived to 14 days.

One potential factor that may account for the different observations among these studies is genetic variability among the strains, which had been collected from diverse geographic locations. Another contributing factor includes the differences in immunosuppression among the model systems. Taken together, it appears that a subset of *C*. *auris* exhibits a high level of virulence. Typically, the virulence of *Candida* in noncompromised infection models is blunted. The unexpected fitness of *C*. *auris* in several immunocompetent models suggests that this species may withstand host immune responses that are typically sufficient to prevent invasive disease caused by other *Candida* spp.

## Is the immune response to *C*. *auris* effective?

**Fig 1:**
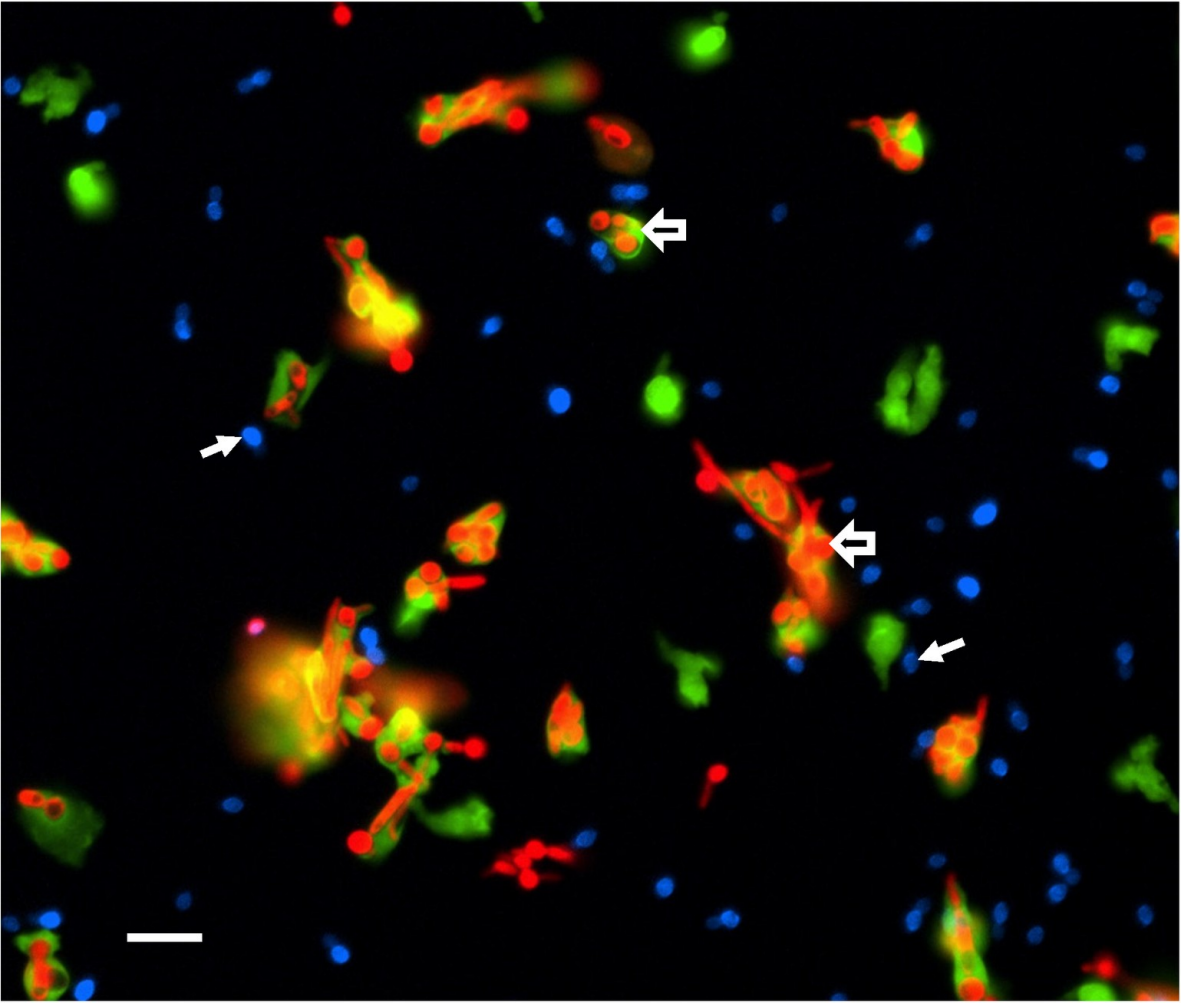
Human neutrophils fail to engage *Candida auris*. Calcein AM-labeled human neutrophils (green) were cocultured with red fluorescent protein-tagged *C*. *albicans* (red) and calcofluor white-stained *C*. *auris* (blue) for 30 min. Neutrophils preferentially engaged *C*. *albicans*, ignoring *C*. *auris*. Open arrows point to neutrophils phagocytosing *C*. *albicans*. Closed arrows show *C*. *auris* cells. Few neutrophils engage *C*. *auris* in the presence or absence of *C*. *albicans*. Measurement bar represents 10 μm.

A recent report questioned the efficacy of the phagocytic response against a *C*. *auris* strain collected from India [28]. Neutrophils, key leukocytes for the control of invasive candidiasis, kill fungi through phagocytosis and the delivery of antimicrobial contents during neutrophil extracellular trap (NET) formation [29, 30]. However, human neutrophils were found to lack effective activity against *C*. *auris*. They failed to engage *C*. *auris*, phagocytose the yeast, or release NETs. When presented with both *C*. *albicans* and *C*. *auris*, human neutrophil exhibited a strong preference for engaging and killing *C*. *albicans* (Fig 1) [28]. The phagocytes functioned normally against *C*. *albicans* but appeared to disregard *C*. *auris*. In vivo analysis of neutrophils in a zebrafish model of invasive candidiasis mimicked the phenotype.

This impaired innate immune response may help to explain why mortality rates remain high even for patients treated with appropriate antifungals [5]. However, little is known regarding the mechanism underlying this phenomenon or how the genetic diversity of *C*. *auris* may influence evasion of neutrophil engulfment. It is intriguing that a similar pattern of phagocyte evasion has been observed for *C*. *lusitaniae*, a species phylogenetically close to *C*. *auris* [31]. Ex vivo studies employing a macrophage cell line found that *C*. *lusitaniae* avoids phagocytosis, in contrast to *C*. *albicans* and *C*. *glabrata*, which are more readily engulfed [32]. The similarity of the phagocytic responses to *C*. *auris* and *C*. *lusitaniae* prompts the question of an altered fungal component that is shared for the two species but divergent from more distantly related *Candida* species. Investigations are also exploring the recognition of *C*. *auris* by mononuclear cells. Peripheral blood mononuclear cells appear to elicit unique cytokine responses upon encounter with *C*. *auris* and *C*. *albicans*, suggesting exposure to different fungal components [33].

## Why are future investigations of *C*. *auris* needed?

Unlike other *Candida* species, *C*. *auris* has emerged as a nosocomial threat, exhibiting rapid person-to-person transmission and causing critical invasive disease. Recent investigations are just beginning to shed light on the mechanisms of pathogenicity for this deadly infection. In several models of invasive disease, strains of *C*. *auris* display virulence similar to that of the most virulent *Candida* species. The observation that neutrophils exhibit reduced activity against *C*. *auris* may contribute to poor outcomes for patients with invasive disease. However, the high mortality for invasive candidiasis likely also reflects the comorbidities and prolonged hospitalizations for this cohort. One promising finding is the efficacy of the *C*. *albicans* NDV-3A vaccine for protection against *C*. *auris* in mice [34]. Prevention would be ideal for the vulnerable population of hospitalized patients.

In light of the considerable diversity among the circulating *C*. *auris* strains, investigations to understand the influence of genetic diversity on immunity will be valuable. Furthermore, recent studies have revealed a filamentous form of *C*. *auris*, which appears to be triggered by a heritable phenotypic switch generated in vivo [35]. With inducing conditions and filamentation regulators distinct from *C*. *albicans*, the impact of this morphological transition on immune cell recognition is a mystery. As the tools for genetic manipulation of *C*. *auris* are becoming available, it will be fascinating to see how these genetic and phenotypic variations influence virulence and immunity [36].
